# Effects of Sodium–Glucose Cotransporter-2 Inhibitors on Anemia in Patients with Chronic Kidney Disease: A Pre–Post Observational Analysis

**DOI:** 10.3390/medsci14020328

**Published:** 2026-06-17

**Authors:** Selena Gajić, Filip Simović, Ana Bontić, Aleksandra Kezić, Milorad Stojadinović, Svetozar Mijušković, Jelena Pavlović, Vidna Karadžić Ristanović, Verica Stanković Popović, Dušan Vićentijević, Milija Bjeličić, Kristina Petrović, Ivana Mrđa, Kristina Filić, Saddam Shawamri, Sanja Stanković, Marko Baralić

**Affiliations:** 1 Clinic of Nephrology, University Clinical Center of Serbia, Pasterova 2, 11000 Belgrade, Serbia; 2 Faculty of Medicine, University of Belgrade, Dr Subotica Starijeg 8, 11000 Belgrade, Serbia; 3 Center for Medical Biochemistry, University Clinical Center of Serbia, 11000 Belgrade, Serbia; 4 Faculty of Medical Sciences, University of Kragujevac, 34000 Kragujevac, Serbia

**Keywords:** chronic kidney disease, anemia, SGLT2 inhibitors, empagliflozin, dapagliflozin, hemoglobin, ferritin

## Abstract

**Background and Objectives**: Anemia is a common complication of chronic kidney disease (CKD) and is associated with reduced quality of life, accelerated disease progression, and increased cardiovascular risk. Sodium–glucose cotransporter-2 inhibitors (SGLT2is) have demonstrated significant renal and cardiovascular benefits, and clinical trials have reported improvements in hematologic parameters during treatment. However, real-world evidence regarding their longitudinal effects on hemoglobin (Hb) and iron metabolism in patients with CKD remains limited. **Materials and Methods**: We conducted a pre–post analysis of 118 adult patients with CKD stages 1–4 treated with SGLT2is (empagliflozin or dapagliflozin) at the University Clinical Center of Serbia between January 2024 and June 2025. Patients received either agent at 10 mg once daily for 18 months. Hb, ferritin, C-reactive protein (CRP), albumin (Alb), daily proteinuria (Prt), and estimated glomerular filtration rate (eGFR) were assessed at baseline and at 18 months. Ferritin was adjusted for inflammatory and nutritional status using a residualization model incorporating CRP and Alb. Changes between the two time points were analyzed using repeated-measures general linear models (GLMs). **Results**: In unadjusted analyses, mean Hb increased modestly from 136.5 ± 17.9 g/L at baseline to 138.8 ± 18.9 g/L at follow-up (*p* = 0.028), while median ferritin decreased from 102.2 µg/L to 89.9 µg/L (*p* = 0.011). After adjustment for CRP and Alb, ferritin levels remained unchanged (*p* = 0.752). Repeated-measures analyses showed no significant longitudinal effect of time on Hb or ferritin and no significant interaction between time and SGLT2i type. Baseline eGFR, Prt, sex, and baseline ferritin significantly influenced longitudinal hematologic trajectories. **Conclusions**: SGLT2i therapy was associated with modest increases in Hb levels over 18 months, while inflammatory status remained stable and no significant reduction in ferritin levels was observed after adjustment for inflammatory and nutritional factors. Longitudinal Hb and ferritin trajectories did not differ significantly between empagliflozin and dapagliflozin, while baseline kidney function, Prt, iron status, and sex significantly influenced hematologic outcomes. Although causal inference is limited by the absence of a control group, these findings suggest a possible favorable effect of SGLT2is on anemia-related parameters in patients with CKD.

## 1. Introduction

Anemia is one of the most common complications in patients with chronic kidney disease (CKD) and contributes substantially to morbidity and mortality in this population [1,2]. It is defined as a hemoglobin (Hb) concentration < 12 g/dL (<120 g/L) in women and <13 g/dL (<130 g/L) in men [3]. It negatively affects quality of life, causing fatigue, weakness, reduced exercise tolerance, cognitive impairment, refs. [4,5] and an increased risk of cardiovascular complications, including left ventricular hypertrophy (LVH) and heart failure (HF). The prevalence and severity of anemia increase as kidney function declines, reflecting impaired erythropoietin (EPO) production, chronic inflammation, and altered iron metabolism [1,2].

Sodium–glucose cotransporter-2 inhibitors (SGLT2is) were initially developed for the treatment of type 2 diabetes mellitus (T2DM), but their use has expanded following evidence of significant renal and cardiovascular benefits [6]. Large randomized clinical trials have demonstrated that SGLT2is reduce the risk of CKD progression and cardiovascular death or hospitalization for heart failure, irrespective of ejection fraction or diabetes status, as well as the risk of acute renal failure [7].

In addition to these cardiorenal benefits, improvements in hematologic parameters, including hemoglobin (Hb) and hematocrit (Hct), have been observed in clinical trials [6,8,9,10]. Several mechanisms have been proposed to explain the effects of SGLT2is on erythropoiesis and anemia [6,11]. Because SGLT2i-induced glucosuria and natriuresis lead to osmotic diuresis, the increase in hematocrit observed with SGLT2is may initially be attributed to hemoconcentration resulting from volume contraction. However, if hemoconcentration were the primary mechanism, it would be expected to be associated with worsening renal function, as observed with long-term diuretic use. In contrast, SGLT2is are associated with long-term preservation of renal function [12]. Instead, there is now consistent evidence that initiation of SGLT2i therapy is accompanied by increased EPO levels, reticulocytosis, and expansion of red blood cell mass, suggesting true stimulation of erythropoiesis rather than simple hemoconcentration [13].

Despite consistent evidence of beneficial effects on anemia-related parameters in randomized trials, data from real-world patients with CKD remain limited, particularly regarding potential differences between individual SGLT2i agents. Therefore, the aim of this study was to evaluate pre–post changes in Hb and iron-related parameters following SGLT2i initiation in patients with CKD stages 1–4 and to compare the effects of empagliflozin and dapagliflozin in a real-world clinical setting. In our cohort, initiation of SGLT2i therapy was guided by contemporary clinical practice guidelines recommending their use for the attenuation of CKD progression and reduction of cardiovascular risk, irrespective of diabetes status [14].

To our knowledge, this represents the first comparative analysis of SGLT2i effects on anemia-related parameters in patients with CKD in the Republic of Serbia.

## 2. Materials and Methods

### 2.1. Study Participants

This prospective pre–post observational study utilized data extracted from the University Clinical Center of Serbia (UCCS) electronic medical record system. The study included 118 adult patients (≥18 years) with CKD stages 1–4 who had a KDIGO guideline-based indication for initiation of SGLT2i therapy. Indications included persistent albuminuria (urine albumin/creatinine ratio (UACR) ≥ 200 mg/g or equivalent proteinuria (Prt)), and/or estimated glomerular filtration rate (eGFR) ≥ 20 mL/min/1.73 m^2^ with evidence of progressive CKD, regardless of diabetes status, in the absence of contraindications.

To minimize selection bias, all consecutive eligible patients meeting KDIGO criteria for SGLT2i initiation and managed at the outpatient nephrology clinic of the UCCS during the inclusion period were enrolled. Accordingly, the study cohort comprised all patients in whom SGLT2i therapy was initiated in January 2024, with prospective follow-up through June 2025. Exclusion criteria included anemia not attributable to CKD, CKD stage 5, hematological malignancy, liver cirrhosis, and treatment with iron supplementation or erythropoiesis-stimulating agents. This design captures a real-world, unselected patient cohort reflective of routine nephrology clinical practice in patients with CKD, thereby reducing the risk of selection bias and enhancing external validity and the generalizability of the findings. The study was conducted and reported in accordance with the STROBE (Strengthening the Reporting of Observational Studies in Epidemiology) statement.

Given the observational nature of the study, treatment allocation was determined by routine clinical decision-making rather than randomization, introducing the possibility of confounding by indication. To address this limitation, all analyses were adjusted for key baseline clinical variables associated with disease severity and hematologic status, including eGFR, Prt, inflammatory markers, and baseline Hb and iron status.

A non-treated control group was not included, as withholding guideline-indicated SGLT2i therapy would have been ethically inappropriate. Accordingly, the study captures within-patient changes across two time points and compares outcomes between empagliflozin and dapagliflozin, without the ability to attribute observed hematologic trajectories causally to SGLT2i therapy. Natural disease progression, secular trends, and concurrent therapies may have contributed to the observed changes and cannot be excluded.

All patients received SGLT2i therapy (empagliflozin or dapagliflozin) at a dose of 10 mg once daily for 18 months, from January 2024 to June 2025, in an outpatient setting at the UCCS Clinic of Nephrology. CKD and its stages were defined according to the Kidney Disease: Improving Global Outcomes (KDIGO) guidelines [14].

Patients were followed at 3–6 month intervals, depending on CKD stage. The study protocol was approved by the Ethics Committee of the UCCS (Decision No. 1322/X-7, dated 10 October 2022) and was conducted in accordance with the Declaration of Helsinki.

Demographic, clinical, and laboratory data were extracted from the UCCS electronic medical records. All patients adhered to a standardized hygienic-dietary regimen, and 86.4% were treated with maximally tolerated doses of renin–angiotensin–aldosterone system inhibitors (RAASis).

### 2.2. Laboratory Tests

Blood and urine samples and blood pressure measurements were obtained from all patients at each nephrology visit, and they were compared for this study at two time points: at the start of SGLT2i treatment and 18 months after. All biochemical samples were collected early in the morning, 12 h after the patient’s last meal. For biochemical analyses, blood was collected in tubes without the addition of anticoagulants. Blood samples were collected from all patients in the central laboratory of the UCCS without storage but were immediately processed. Biochemical parameters, such as glycemia, serum creatinine, eGFR, serum albumin (Alb), ferritin, transferrin saturatin (TSAT), C-reactive protein (CRP) and 24 h Prt were determined using routine laboratory test procedures on an automated analyzer Architect ci8200 (Abbott Diagnostics, Wiesbaden, Germany).

### 2.3. Statistical Analyses

Data were numerically coded, tabulated, and checked for completeness prior to statistical analysis. Continuous variables were expressed as mean ± standard deviation for normally distributed data or median [interquartile range] for non-normally distributed data, while categorical variables were reported as frequencies and percentages. Normality was assessed using appropriate distribution tests to determine appropriate statistical tests, parametric or non-parametric. Between-group comparisons were performed using the independent samples *t*-test or Mann–Whitney U test, as appropriate. Within-group longitudinal changes were analyzed using paired *t*-test or Wilcoxon signed-rank test, depending on distribution. Categorical variables were analyzed using the chi-square or Fisher’s exact test, depending on expected cell counts.

The primary objective was to evaluate the longitudinal effects of empagliflozin and dapagliflozin on Hb and iron-related parameters. Serum ferritin was used as a surrogate marker of iron stores; however, given its known behavior as an acute-phase reactant, values were adjusted for systemic inflammation and nutritional status using a residualization approach based on CRP and Alb, as previously described by McSorley et al. [15], according to the following equation:

ln Feradj = ln Ferobs − (β1(ln CRPobs − ln CRPref)) − (β2(ln Albobs − ln Albref))

Legend: ln Feradj—natural logarithm of adjusted ferritin; ln Ferobs—natural logarithm of observed ferritin; ln CRPobs—natural logarithm of observed CRP; ln CRPref—natural logarithm of reference CRP; ln Albobs—natural logarithm of observed Alb; ln Albref—natural logarithm of reference Alb; β1, β2—regression coefficients representing the effect of CRP and Alb on ferritin levels, respectively.

The calculated adjusted ferritin values were then back-transformed to obtain the adjusted ferritin estimates, although for further statistical analysis log-transformed value was used because of non-normal distribution. The reference value for Alb was 40 g/L and CRP 1.3 mg/L.

This approach accounts for the known effect of inflammatory markers on ferritin levels, as even low levels of inflammation have been shown to significantly influence serum ferritin concentrations [13].

Longitudinal changes in ferritin and Hb over 18 months were analyzed using General Linear Models with repeated measures, with time (baseline vs. 18 months) as the within-subject factor and SGLT2i type (empagliflozin vs. dapagliflozin) as the between-subject factor. Models were adjusted for clinically relevant covariates, including age, sex, baseline eGFR, Prt, RAASi use, and baseline values of the dependent variable (with reciprocal adjustment of ferritin and Hb where appropriate).

Effect sizes were reported as partial eta squared (η^2^), and estimated marginal means were calculated with Bonferroni correction for multiple comparisons. Given the relatively small sample size and observational design, emphasis was placed on estimation-based interpretation, including effect sizes and model-derived estimates, rather than solely on *p*-values.

Model assumptions, including normality, homogeneity of variances, and equality of covariance matrices, were evaluated. Sphericity was inherently satisfied due to the presence of only two time points. Levene’s test assessed homogeneity of variances, while Box’s M test evaluated equality of covariance matrices. In cases of violation of the latter, multivariate inference was based on Pillai’s Trace, which provides a more robust estimate under assumption violations.

All statistical analyses were performed using SPSS version 27 (IBM Corp., Armonk, NY, USA) and G*Power version 3.1.9.7 for power analysis, with a two-sided significance level set at α = 0.05.

## 3. Results

Among patients included in the analysis, the median age was 68 years [55; 75]. Median Prt was 0.20 g/24 h [0.10; 0.78] at baseline and remained unchanged at follow-up 0.20 g/24 h [0.10; 0.51], with a median Prt reduction of 0.00 g/24 h [−0.16; 0.05]. Median eGFR was 37.5 mL/min per 1.73 m^2^ [26.0; 49.3]. Mean Hb increased modestly from 136.5 ± 17.9 g/L at baseline to 138.8 ± 18.9 g/L at follow-up. The median change in Hb was 2.0 g/L [−4.3; 9.0]. Transferrin saturation (TSAT) demonstrated a minimal increase, from a median of 27.5% [21.0; 37.0] to 28.0% [20.3; 36.0]. The median change in ferritin was −0.03 [−0.35; 0.48] (Table 1 and Table 2).

Among the 118 patients included in the cohort, 62 (52.5%) were female and 56 (47.5%) were male. The most common primary kidney disease was hypertension (HTN) (45 patients, 38.1%), followed by T2DM (32 patients, 27.1%) and glomerulonephritis (GN) (26 patients, 22.0%), while other causes were present in 15 patients (12.7%). T2DM was present in 41 patients (34.7%), and the majority of patients had HTN (107 patients, 90.7%). Most patients were receiving RAASi therapy (102 patients, 86.4%). Regarding SGLT2i treatment, 71 patients (60.2%) received empagliflozin and 47 patients (39.8%) received dapagliflozin (Table 3).

There was no significant difference in age between patients treated with empagliflozin and dapagliflozin (69 [58; 74] vs. 67 [52; 75] years, *p* = 0.721). Baseline Prt was numerically lower in the empagliflozin group, although this difference did not reach statistical significance (0.16 [0.10; 0.60] vs. 0.30 [0.13; 1.75] g/24 h, *p* = 0.086). Prt at follow-up was similar between groups (0.18 [0.10; 0.40] vs. 0.20 [0.12; 0.70] g/24 h, *p* = 0.518). However, the median reduction in Prt differed between groups (0.00 [−0.10; 0.09] vs. −0.04 [−0.41; 0.00] g/24 h, *p* = 0.017). Median eGFR did not differ significantly between the empagliflozin and dapagliflozin groups (35 [25; 47] vs. 42 [28; 52] mL/min per 1.73 m^2^, *p* = 0.112). During the 18-month follow-up, 34 patients (28.8%) changed their CKD stage, with five patients (14.7%) progressing by two stages (Table 4).

Mean Hb levels were lower in the empagliflozin group compared with the dapagliflozin group both at baseline (133.5 ± 16.2 vs. 140.9 ± 19.4 g/L, *p* = 0.028) and at follow-up (134.4 ± 16.6 vs. 145.4 ± 20.3 g/L, *p* = 0.002). Serum Alb was slightly lower in the empagliflozin group at baseline (44 [41; 46] vs. 45 [43; 47] g/L, *p* = 0.046), while no significant difference was observed at follow-up (*p* = 0.336). No significant between-group differences were observed in ferritin, TSAT or CRP levels at baseline or follow-up (all *p* > 0.05) (Table 4).

There was no significant difference in sex distribution between the empagliflozin and dapagliflozin groups (female: 54.8% vs. 45.2%; male: 66.1% vs. 33.9%; *p* = 0.291). The prevalence of T2DM did not differ significantly between treatment groups (*p* = 0.211), nor did the prevalence of HTN (*p* = 0.522) or use of RAASi (*p* = 0.115). The distribution of primary kidney disease differed significantly between groups (*p* = 0.023). Hypertensive nephropathy was more frequent in the empagliflozin group (71.1% vs. 28.9%), whereas GN was more common in the dapagliflozin group (57.7% vs. 42.3%). The proportion of patients with diabetic kidney disease was similar between groups (50.0% vs. 50.0%) (Table 5).

In unadjusted paired analyses (paired *t*-test or Wilcoxon signed-rank test, as appropriate), median ferritin levels decreased from 102.20 µg/L [45.30; 190.25] at baseline to 89.90 µg/L [48.0; 157.1] at 18 months (*p* = 0.011). However, after adjustment for CRP and Alb, ferritin levels did not differ significantly between baseline and follow-up (67.65 µg/L [34.3; 151.9] vs. 71.09 µg/L [34.8; 135.1] (*p* = 0.752). Median CRP levels remained stable over time, with no significant difference between baseline and 18 months (3.50 mg/L [1.58; 6.73] vs. 2.90 mg/L [1.58; 6.6], *p* = 0.497). In contrast, serum Alb levels increased modestly from 44.00 g/L [42; 47] to 45.00 g/L [43; 47] (*p* = 0.001). Mean Hb levels increased slightly from 136.5 ± 17.9 g/L at baseline to 138.8 ± 18.9 g/L at 18 months (*p* = 0.028) (Table 1).

In repeated-measures GLM analysis, there was no significant overall effect of time on ferritin (F = 0.006, *p* = 0.940, partial η^2^ < 0.01) or Hb levels (F = 1.34, *p* = 0.250, partial η^2^ = 0.012) over 18 months. Similarly, there was no significant time × SGLT2i interaction for ferritin (F = 0.001, *p* = 0.980, partial η^2^ < 0.01) or Hb (F = 3.26, *p* = 0.074, partial η^2^ = 0.030), indicating no differential longitudinal effect between empagliflozin and dapagliflozin. There was no significant overall group effect of SGLT2i type on ferritin levels (F = 0.18, *p* = 0.670, partial η^2^ = 0.002). However, Hb levels differed between treatment groups independent of time (F = 4.53, *p* = 0.036, partial η^2^ = 0.041) (Table 6).

Significant interaction effects were observed between time and baseline renal function as well as baseline Prt on longitudinal changes in hematologic parameters, Hb and ferritin. Specifically, the time × baseline eGFR interaction was significant for both Hb (F = 12.01, *p* = 0.001, partial η^2^ = 0.101) and ferritin (F = 4.03, *p* = 0.047, partial η^2^ = 0.036), indicating that the magnitude of change in these parameters over 18 months varied according to baseline kidney function. Similarly, the time × baseline Prt interaction was significant for Hb (F = 5.26, *p* = 0.024, partial η^2^ = 0.047) and ferritin (F = 5.76, *p* = 0.018, partial η^2^ = 0.051), suggesting that the baseline Prt also modified the longitudinal trajectory of these parameters (Table 6).

These interaction effects indicate that the longitudinal trajectories of Hb and ferritin were not uniform across the cohort but depended on baseline kidney function and Prt. Specifically, patients with differing levels of baseline eGFR and Prt exhibited heterogeneous changes in hematologic parameters, Hb and ferritin, over time, suggesting that CKD severity modifies the hematologic response associated with SGLT2i therapy.

Sex was also significantly associated with both ferritin (F = 12.02, *p* = 0.001, partial η^2^ = 0.101) and Hb levels (F = 18.09, *p* < 0.001, partial η^2^ = 0.145). In addition, baseline ferritin significantly modified longitudinal Hb changes (F = 4.04, *p* = 0.047, partial η^2^ = 0.036) (Table 6).

All assumption checks confirmed model validity for ferritin analyses, with no violations of multivariate or variance homogeneity assumptions. For Hb, Box’s M was significant, so results were interpreted using Pillai’s Trace. All other model assumptions were also met (Table 6).

Repeated-measures GLM showed no significant change in TSAT over time, no time × SGLT2i interaction, and no overall between-group difference. Baseline Hb was the only variable significantly associated with TSAT, while no other covariates were significantly associated. Model assumptions were satisfied. Details are presented in Table 7.

**Figure 1:**
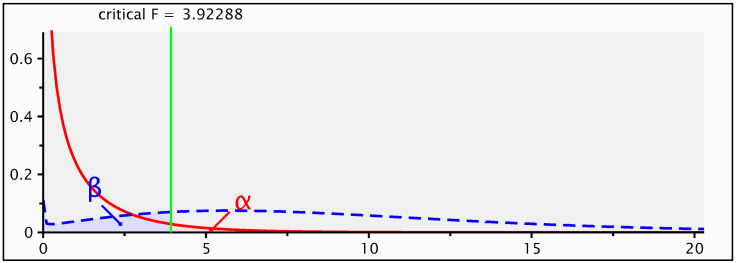
Post hoc sensitivity analysis (G*Power) indicating minimum detectable effect size.

A post hoc sensitivity analysis indicated that the study was powered (80%) to detect small-to-moderate interaction effects (Cohen’s f ≈ 0.13). The observed effect sizes for longitudinal changes in Hb and ferritin were smaller than this threshold, suggesting that any undetected effects are likely to be of minimal clinical relevance rather than indicating a type II error due to insufficient sample size, as presented in Figure 1.

## 4. Discussion

In this pre–post analysis of patients with CKD stages 1–4 initiated on SGLT2i, Hb levels increased modestly over 18 months in unadjusted analyses; however, this effect was not confirmed in repeated-measures GLMs after adjustment for relevant covariates. Similarly, ferritin levels decreased in unadjusted paired comparisons but did not remain significantly different after adjustment for inflammatory and nutritional markers. Furthermore, no significant time × treatment interaction was observed for either Hb or ferritin, indicating comparable longitudinal trajectories in patients treated with empagliflozin and dapagliflozin. Notably, baseline clinical characteristics, particularly kidney function, Prt, iron status, and sex, emerged as the principal determinants of hematologic trajectories during follow-up. These findings suggest that the severity and underlying characteristics of CKD may exert a greater influence on anemia-related parameters than the specific SGLT2i agent used.

The absence of a non-treated control group precludes causal inference regarding the hematologic effects of SGLT2i therapy. Consequently, the observed changes may reflect a combination of treatment-related effects, underlying disease progression, changes in concomitant therapy, and regression to the mean. Nevertheless, the consistent influence of baseline eGFR, Prt, iron status, and sex across models highlights the importance of patient-related factors in shaping hematologic outcomes during long-term follow-up.

Previous systematic reviews and meta-analyses have shown that Hb and Hct levels significantly increased with SGLT2i therapy, including empagliflozin, dapagliflozin, and canagliflozin, although a key limitation of these reports is that hematologic changes were not the primary prespecified outcomes [8]. Treatment with SGLT2is has been shown to increase hematocrit by 2% to 5% and raise Hb levels by 0.4 to 1.0 g/dL compared with placebo across all SGLT2is, with empagliflozin showing the highest increase in Hct and dapagliflozin being the only SGLT2i with a dose-dependent effect [9,16]. Initially, the increase in Hb and Hct levels was attributed to hemoconcentration due to the diuretic effect of these agents, although it soon became apparent that these drugs increased EPO levels and erythropoiesis, and improved iron metabolism. Initial increase in EPO levels and/or reticulocyte counts was observed with the use of these agents, both in patients with either normal or reduced renal function, confirming their direct positive effect on erythropoiesis. Recently, in a post hoc analysis of the Dapagliflozin on Renal Outcomes and cardiovascular Mortality in Patients With Chronic Kidney Disease (DAPA-CKD) study, it was found that the increase in Hct was gradual and reached a maximum about four months after the initiation of the treatment with dapagliflozin, which rules out its relationship with the initial decrease in blood volume [17,18]. However, these studies were conducted in controlled trial settings with selected patient populations.

SGLT2i erythropoiesis effects have been proposed to result from complex pathophysiological mechanisms, including enhanced EPO production via reduced renal cortical hypoxia, suppression of inflammation, activation of hypoxia-inducible factor (HIF) pathways, hepcidin suppression, and improved iron mobilization [19]. It must be acknowledged, however, that none of these mechanistic intermediates—EPO, hepcidin, or reticulocyte count—were measured in the present study because these assays were not routinely available at our institution, reflecting resource constraints typical of many middle-income healthcare settings. Accordingly, the mechanistic discussion presented here should be read as contextual background rather than as an interpretation of our own findings.

In contrast to the results of randomized clinical trials [8], our adjusted pre–post analyses did not demonstrate a significant independent time effect on Hb. This discrepancy may reflect differences between randomized trial populations and real-world CKD cohorts. Our study included patients with heterogeneous primary kidney diseases, variable kidney function, and differing baseline iron status, all of which significantly influenced Hb trajectories. In particular, baseline eGFR and Prt significantly modified hematologic changes between the two time points. The observed interaction between time and baseline eGFR, as well as Prt, suggests that hematologic trajectories observed following SGLT2i initiation are influenced by underlying CKD severity. This finding is biologically plausible, as reduced eGFR reflects impaired renal oxygen sensing and EPO production, while higher levels of Prt are associated with systemic inflammation and altered iron metabolism [20,21,22]. SGLT2is have been shown to improve renal cortical oxygenation and stimulate erythropoiesis, potentially through modulation of HIF pathways [23,24,25]. Therefore, patients with more advanced CKD or higher Prt may exhibit a differential hematologic response over time, reflecting variability in baseline pathophysiology. These findings are consistent with established evidence that CKD severity is a major determinant of anemia progression, independent of pharmacologic therapy [26]. Reduced kidney function is associated with impaired EPO production, inflammation, and altered iron utilization, all of which may attenuate treatment-related effects [27,28].

In the repeated-measures GLM analysis, no significant overall effect of time on Hb levels was observed, nor was there a significant time × treatment interaction, indicating that longitudinal Hb trajectories did not differ significantly between empagliflozin and dapagliflozin. Although a significant overall group effect of SGLT2i type was detected, this finding should be interpreted with caution given the non-randomized study design and baseline imbalances between treatment groups. Specifically, the lower baseline Hb levels and higher prevalence of hypertensive nephropathy in the empagliflozin group, together with the significant difference in the distribution of primary kidney disease between groups (*p* = 0.023), suggest that the observed group effect may reflect underlying patient characteristics rather than a true treatment-specific effect. In contrast, baseline eGFR, Prt, sex, and ferritin significantly influenced Hb trajectories over time, indicating that underlying disease severity and iron status were more important determinants of hematologic outcomes than the specific SGLT2i agent used.

Ferritin levels decreased in unadjusted analyses but did not differ significantly after adjustment for CRP and Alb levels. This is consistent with the dual role of ferritin as both an iron storage marker and an acute-phase reactant [29]. The correction of ferritin values for inflammatory and nutritional markers has been shown to improve the interpretation of iron status, particularly in chronic disease populations [30]. The absence of an adjusted longitudinal ferritin change in our study suggests that apparent reductions in ferritin may reflect changes in inflammatory or nutritional status rather than true alterations in iron stores.

Sex was independently associated with both Hb and ferritin levels, consistent with well-established physiologic differences in erythropoiesis and iron metabolism. In addition, baseline ferritin significantly modified longitudinal Hb trajectories, supporting the importance of baseline iron status as a determinant of erythropoietic response [30,31]. These findings align with real-world observational studies demonstrating that baseline clinical characteristics play a central role in determining hematologic responses to SGLT2i therapy [24].

During the 18-month follow-up, kidney function improved, with median eGFR increasing from 37.5 [26.0; 49.3] to 40.2 [25.0; 62.7] mL/min/1.73 m^2^, while Prt decreased significantly despite stable median values (eGFR: *p* < 0.001; Prt *p* = 0.031). Kidney function was comparable between treatment groups. Most patients were on RAASis, reflecting contemporary CKD management.

This study has several important limitations. The absence of a non-treated control group is the most significant, as it precludes causal inference regarding the hematologic effects of SGLT2i therapy. Without a parallel untreated CKD cohort, it is not possible to distinguish treatment-related effects from the natural course of CKD-associated anemia, regression to the mean, changes in concomitant therapies, or other unmeasured factors. In addition, baseline imbalances between treatment groups, particularly in Hb levels and the distribution of primary kidney diseases, may have influenced between-group comparisons.

The sample size was relatively modest for a repeated-measures GLM incorporating multiple covariates, and no formal a priori power calculation was performed. Furthermore, key mechanistic intermediates, including EPO, hepcidin, and reticulocyte count, were not measured because these assays were not routinely available at our institution, limiting mechanistic interpretation of the observed hematologic changes.

HbA1c data were not systematically available for all participants, particularly those without diabetes. However, glycemia was monitored as part of routine clinical care, and fasting glucose levels remained stable throughout follow-up, suggesting that changes in glycemic control were unlikely to have substantially confounded the hematologic outcomes.

Although analyses stratified by CKD stage might have reduced clinical heterogeneity, such an approach would have substantially decreased statistical power because of the limited sample size within individual CKD subgroups.

Despite these limitations, the present study provides real-world longitudinal data from a heterogeneous and unselected CKD cohort managed according to contemporary clinical practice guidelines. The consistent influence of baseline eGFR, Prt, iron status, and sex on hematologic trajectories highlights the importance of patient-related factors in shaping anemia-related outcomes during follow-up. These findings suggest that the hematologic response observed during SGLT2i therapy may be substantially modified by underlying clinical characteristics and CKD severity.

## 5. Conclusions

In this cohort of patients with CKD stages 1–4, SGLT2i therapy was associated with modest increases in Hb levels over 18 months in unadjusted analyses, while inflammatory status remained stable. No significant reduction in ferritin levels was observed after adjustment for inflammatory and nutritional factors. Longitudinal Hb and ferritin trajectories did not differ significantly between the empagliflozin and dapagliflozin treatment groups. Baseline kidney function, 24 h Prt, iron status, and sex significantly influenced hematologic outcomes over time, suggesting that CKD severity may modify the hematologic response to SGLT2i therapy. Although the prospective design strengthens the assessment of longitudinal changes, the absence of a control group and randomization precludes definitive conclusions regarding causality. Nevertheless, these findings suggest a possible favorable effect of SGLT2i on anemia-related parameters in patients with CKD. Further prospective controlled studies are warranted to confirm these observations and clarify the underlying mechanisms.

## Figures and Tables

**Table 1:** Demographic data and unadjusted paired comparisons of ferritin (unadjusted and adjusted for CRP and albumin), CRP, albumin, and hemoglobin between baseline and 18-month follow-up. Continious variables (n = 118). *p* values were calculated using paired *t*-tests or Wilcoxon signed-rank tests, as appropriate.

Variable	T1 Mean ± SD/Median [Q1; Q3]	T2 Mean ± SD/Median [Q1; Q3]	*p*-Value
Age (years)	68 [55; 75]	-	-
eGFR (mL/min/1.73 m^2^)	37.5 [26.0; 49.3]	40.2 [25.0; 62.7]	<0.001
Prt (g/24 h)	0.20 [0.10; 0.78]	0.20 [0.10; 0.51]	0.031
Hb (g/L)	136.5 ± 17.9	138.8 ± 18.9	0.028
TSAT (%)	27.5 [21.0; 37.0]	28.0 [20.3; 36.0]	0.915
Ferritin (unadjusted)	102.20 [45.30; 190.25]	89.90 [48.0; 157.1]	0.011
Ferritin (adjusted)	67.65 [34.3; 151.9]	71.09 [34.8; 135.1]	0.752
CRP (mg/L)	3.50 [1.58; 6.73]	2.90 [1.58; 6.6]	0.497
Alb (g/L)	44.00 [42; 47]	45.00 [43; 47]	0.001

T1—baseline measure; T2 measure after 18 months; Prt—proteinuria; Hb—hemoglobin; eGFR—estimated glomerular filtration rate; TSAT—transferrin saturation; CRP—C-reactive protein; Alb—albumin. Data are presented as mean ± SD or median [Q1; Q3], as appropriate.

**Table 2:** Changes in laboratory parameters over 18 months.

Variable	Median [Q1; Q3]
ΔPrt (g/24 h)	0.00 [−0.16; 0.05]
ΔHb (g/L)	2.0 [−4.3; 9.0]
ΔFerritin (µg/L)	−0.03 [−0.35; 0.48]
ΔTSAT (%)	0.0 [−8.0; 6.0]
ΔCRP (mg/L)	0.0 [−1.9; 1.1]
ΔAlb (g/L)	1.0 [−1.0; 3.0]
ΔeGFR (mL/min/1.73 m^2^)	3.85 [−5.78; 14.85]

Prt—proteinuria; Hb—hemoglobin; TSAT—transferrin saturation; CRP—C-reactive protein; Alb—albumin; eGFR—estimated glomerular filtration rate. Δ indicates change from baseline to the 18-month follow-up.

**Table 3:** Demographic data. Categorical variables (n = 118).

Variable	Category	n %
Sex	female	62 (52.5%)
	male	56 (47.5%)
Primary kidney disease	T2DM	32 (27.1%)
	HTN	45 (38.1%)
	GN	26 (22.0%)
	Other	15 (12.7%)
T2DM	No	77 (65.3%)
	Yes	41 (34.7%)
HTN	No	11 (9.3%)
	Yes	107 (90.7%)
RAASis	No	16 (13.6%)
	Yes	102 (86.4%)
SGLT2is	Empagliflozin	71 (60.2%)
	Dapagliflozin	47 (39.8%)

T2DM—type 2 diabetes mellitus; HTN—hypertension; RAASi—renin–angiotensin–aldosterone system inhibitor; SGLT2i—sodium–glucose cotransporter-2 inhibitors; GN—glomerulonephritis.

**Table 4:** Baselin demographics and longitudinal laboratory data according to SGLT2i treatment group: continuous variables. Group comparisons performed using independent *t*-test or Mann–Whitney U test, as appropriate.

Variable	Empagliflozin Mean ± SD/Median [Q1; Q3]	Dapagliflozin Mean ± SD/Median [Q1; Q3]	*p*-Value
Age (years)	69 [58; 74]	67 [52; 75]	0.721
Prt T1 (g/24 h)	0.16 [0.10; 0.60]	0.30 [0.13; 1.75]	0.086
Prt T2 (g/24 h)	0.18 [0.10; 0.40]	0.20 [0.12; 0.70]	0.518
ΔPrt (g/24 h)	0.00 [−0.10; 0.09]	−0.04 [−0.41; 0.00]	0.017
eGFR (mL/min/1.73 m^2^)	35 [25; 47]	42 [28; 52]	0.112
Hb T1 (g/L)	133.5 ± 16.2	140.9 ± 19.4	0.028
Hb T2 (g/L)	134.4 ± 16.6	145.4 ± 20.3	0.002
ΔHb (g/L)	2.0 [−4.0; 8.0]	1.0 [−6.0; 10.0]	0.603
Ferritin T1 (µg/L)	122.9 [45.4; 188.0]	82.8 [45.0; 200.7]	0.926
Ferritin T2 (µg/L)	85.8 [41.7; 157.0]	95.9 [58.5; 157.2]	0.445
ΔFerritin (µg/L)	−8.4 [−56.8; 9.5]	−5.3 [−23.7; 22.9]	0.141
TSAT T1 (%)	27.0 [19.8; 36.3]	28.0 [21.0; 38.0]	0.503
TSAT T 2 (%)	28.0 [19.8; 36.0]	31.0 [22.8; 38.3]	0.202
ΔTSAT (%)	−1.0 [−8.3; 4.0]	1.5 [−7.3; 8.0]	0.222
CRP T1 (mg/L)	3.7 [1.7; 7.6]	3.2 [1.4; 5.5]	0.360
CRP T2 (mg/L)	2.94 [1.7; 6.6]	2.70 [1.1; 6.6]	0.634
ΔCRP (mg/L)	0.0 [−2.6; 1.1]	0.0 [−1.9; 1.1]	0.737
Alb T1 (g/L)	44 [41; 46]	45 [43; 47]	0.046
Alb T2 (g/L)	45 [43; 47]	46 [44; 48]	0.336
ΔAlb (g/L)	1.0 [−1.0; 3.0]	0.0 [−1.0; 3.0]	0.121

Prt—proteinuria; Hb—hemoglobin; T1—baseline measure; T2 measure after 18 months; eGFR—estimated glomerular filtration rate; CRP—C-reactive protein; Alb—albumin; TSAT—transferrin saturation. Data are presented as mean ± SD or median [Q1; Q3], as appropriate. Δ indicates change from baseline to the 18-month follow-up.

**Table 5:** Baseline patient characteristics by treatment group: categorical variables. Group comparisons were performed using χ^2^ test or Fisher’s exact test.

Variable	Category	Empagliflozin n (%)	Dapagliflozin n (%)	*p*-Value
Sex	female	34 (54.8%)	28 (45.2%)	0.291
	male	37 (66.1%)	19 (33.9%)	
Primary kidney disease	T2DM	16 (50.0%)	16 (50.0%)	0.023
	HTN	32 (71.1%)	13 (28.9%)	
	GN	11 (42.3%)	15 (57.7%)	
	Other	12 (80.0%)	3 (20.0%)	
T2DM	No	50 (64.9%)	27 (35.1%)	0.211
	Yes	21 (51.2%)	20 (48.8%)	
HTN	No	8 (72.7%)	3 (27.3%)	0.522
	Yes	63 (58.9%)	44 (41.1%)	
RAASis	No	13 (81.3%)	3 (18.8%)	0.115
	Yes	58 (56.9%)	44 (43.1%)	

T2DM—type 2 diabetes mellitus; HTN—hypertension; RAASi—renin–angiotensin–aldosterone system inhibitors; GN—glomerulonephritis.

**Table 6:** Repeated-measures GLM analysis of ferritin and hemoglobin over 18 months.

Effect	Outcome	F (1.107)	*p*-Value	Partial η^2^
Time (baseline to 18 months)	Ferritin	0.006	0.940	<0.01
	Hb	1.34	0.250	0.012
Time × SGLT2is	Ferritin	0.001	0.980	<0.01
	Hb	3.26	0.074	0.030
SGLT2is (Group effect)	Ferritin	0.18	0.670	0.002
	Hb	4.53	0.036	0.041
Time × Baseline Prt	Ferritin	5.76	0.018	0.051
	Hb	5.26	0.024	0.047
Time × Baseline eGFR	Ferritin	4.03	0.047	0.036
	Hb	12.01	0.001	0.101
Sex	Ferritin	12.02	0.001	0.101
	Hb	18.09	<0.001	0.145
Time × Baseline ferritin	Hb	4.04	0.047	0.036

Prt—proteinuria; Hb—hemoglobin; eGFR—estimated glomerular filtration rate; SGLT2i—sodium–glucose cotransporter-2 inhibitors.

**Table 7:** Repeated-measures GLM analysis of TSAT over 18 months.

Effect	F (df = 1.97)	*p*-Value	Partial η^2^
Time	0.851	0.359	0.009
Time × SGLT2is	2.066	0.154	0.021
SGLT2is (Group effect)	0.250	0.618	0.003
Time × Baseline Prt	0.062	0.804	0.001
Time × Baseline eGFR	0.683	0.411	0.007
Time × Age	2.179	0.143	0.022
Sex	0.080	0.777	0.001
Baseline Hb	4.902	0.029	0.048

Prt—proteinuria; Hb—hemoglobin; eGFR—estimated glomerular filtration rate; SGLT2i—sodium–glucose cotransporter-2 inhibitor.

## Data Availability

The original contributions presented in this study are included in the article. Further inquiries can be directed to the corresponding author.

## Abbreviations

The following abbreviations are used in this manuscript:

SGLT2i	Sodium–glucose cotransporter-2 inhibitor
CKD	Chronic kidney disease
Hb	Hemoglobin
CRP	C-reactive protein
Alb	Albumin
eGFR	Estimated glomerular filtration rate
GLM	General linear model
LVH	Left ventricular hyperthrophy
HF	Heart failure
EPO	Erythropoietin
T2DM	Type 2 diabetes mellitus
Hct	Hematocrit
UCCS	University Clinical Center of Serbia
KDIGO	Kidney Disease Improving Global Outcomes
RAASi	Renin–angiotensin–aldosterone system inhibitors
Prt	Proteinuria
HTN	Hypertension
GN	Glomerulonephritis
HIF	Hypoxia-inducible factor
UACR	Urine albumin/creatinine ratio
TSAT	Transferrin saturation
